# Lectures Illustrative of Certain Local Nervous Affections

**Published:** 1837-07-01

**Authors:** 


### LECTURES ILLUSTRATIVE OF CERTAIN LOCAL NERVOUS AFFECTIONS. By Sir BENJAMIN C. BRODIE, Bart. F.R.S., Serjeant Surgeon to the King, and Surgeon to St. George's Hospital.

[Concluded from the last Number.]

IN the last number of this Journal, we presented a full account of the two first Lectures contained in the volume before us. They were devoted to the laws of local nervous affections, and to the various forms which they affect. We were unable to enter on the analysis of the third lecture, and were compelled to defer it till the present opportunity.

### Pathology of Hysteria.

It is to this that Sir Benjamin firsts adverts, and we discuss a question which must naturally interest us, and is practically an important one. Do the varied and heterogeneous symptoms which have passed before us, admit of being referred to a common cause, and is that common cause the same state of system which gives rise to the ordinary fits of hysteria?

This question may readily be answered in the affirmative, and more particularly for the following reasons: 1, because patients affected with these local nervous symptoms will, in almost every case, be found to have suffered more or less from hysteria; 2, because a wide acquaintance with these cases, will shew them linked together and constituting a connected series; 3, because morbid anatomy exhibits in all the same negative results.

It is evident, of course, that the source of the symptoms must be sought in some peculiar condition of the nervous system. What is it? Dissection does little to solve the riddle, and the convulsions of hysteria, like those of chorea, and like the spasms of tetanus and of hydrophobia, are not found to depend on such alterations of the brain, of the chord, or of the nerves, as admit of appreciation by our senses.

"I have, in several instances," observes Sir Benjamin, "examined the parts to which hysterical pains had been referred; and in one very aggravated case of the kind, I made a careful dissection of all the nerves by which they were supplied, but I have never been able to discover in them any thing different from what belonged to their natural condition. But every part of the body has its corresponding point in the brain, and the greater number of them have their corresponding points in the spinal chord also. Does the examination of these organs lead to any more satisfactory result? The best proof that it does not do so is furnished by the following circumstance: although so many die of other diseases, who have suffered from hysteria also, and the opportunities of examining the bodies of hysterical patients after death are therefore sufficiently numerous, yet the works of the best morbid anatomists contain no observations whatever on the subject. I have had the opportunity of instituting *post mortem* examinations in three cases, in which the hysterical affections were of so aggravated a kind as to be, directly or indirectly, the cause of death; and you shall know the result. In one of them, the patient laboured under a very severe hysterical pain in the side, and was liable, among various other hysterical symptoms, to fits, in which she was scarcely conscious of her own actions. It must have been in one of these attacks that a great number of needles were introduced into one of her legs, which afterwards occasioned much inflammation and effusion of serum into the cellular texture. The patient died, and the body was most carefully examined, but no morbid appearances of any kind could be discovered in it, except what belonged to the œdematous state of the leg. Another case is one to which I have referred already, in which, the patient having long laboured under an hysterical retention of urine, the bladder was found enormously distended, of a black colour, the mucous membrane and muscular tunic being at the same time much attenuated. This patient was an unmarried female, twenty-nine years of age. Having been previously indisposed for a considerable time, she was supposed to have sprained her wrist in lifting a heavy saucepan. From this time she was never free from pain, in the situation of the outer part of the lower extremity of the radius. The pain extended up the fore-arm, and downwards on the side. In November, 1814, about a month after the occurrence of the accident, she was admitted into the hospital. At this time the most careful examination could detect no alteration in the appearance of the limb, but she complained of a constant and intense pain, which extended from the supposed seat of the injury downwards to the fingers, upwards to the shoulder, and again downwards to the spine and sternum. She had great oppression and difficulty of respiration, occasional twitches of the muscles of the face, and any sudden motion of the hand aggravated all these symptoms, and then threw her into a state approaching to that of syncope; in which she was almost unconscious of all that happened, lying with her eyes wide open, and at last recovering with an hysterical sobbing. Her pulse was feeble, beating 120 times in a minute. Forty ounces of urine were drawn off from the bladder, but without any relief as to the other symptoms. The tongue became black and dry; the pulse more feeble; the belly tympanitic; the alvine evacuations being of a dark colour. Then there was hiccough and vomiting: she became weaker and weaker, and died after the lapse of fourteen days from the time of her admission into the hospital. After death the brain and the thoracic and abdominal viscera were very carefully examined, but no morbid appearances were discovered in any one of them, with the exception of the peculiar condition of the bladder, which was described formerly, and two ulcers of the mucous membrane of the *ileum*, each not more than half an inch in length, but occupying almost the entire circumference of the intestine.

The female, who was the subject of the third case had laboured under a paralytic affection of the lower limbs *(paraplegia)*, which Dr. Seymour believed, with good reason, to be connected with, and the consequence of, hysteria. A practitioner whom she consulted, however, thought it advisable to have recourse to repeated blood-letting and other methods of depletion. The result was, the formation of extensive sloughs of the nates and of the soft parts covering the ankles. The patient was now admitted into the hospital, in a state of great exhaustion, and soon afterwards died. The brain and spinal chord were most carefully examined, in the presence of many of you who are now present, but it could not be discovered that they differed, in the smallest degree, from their natural condition; nor were there any signs of disease in the thoracic or abdominal viscera." 69.

If the nervous system in hysterical patients presents no appreciable change of organization, it does not therefore follow that it is absolutely sound. It would be absurd to suppose that it is constituted precisely as it is in healthy females, although we must admit our incapability of forming any definite ideas upon the subject. Our author supposes that an imperfect development of it may lay the foundation of aggravated hysterical affections, and on such a hypothesis he thinks we may explain the connexion of hysteria with the habits of early life—its prevalence in particular families—and the difficulty of totally eradicating it after it has once been established in the system.

It appears to be certain that whatever may be the condition of the nervous system which disposes the patient to become affected with hysteria, the latter is developed under the influence of causes which impair the bodily health. Whilst the female continues strong and healthy, no hysterical symptoms appear; but if she is weakened by bodily illness, by debilitating treatment, or by moral agencies of a depressing character, hysterical affections are the consequence, and they attack one part of the body or another, as circumstances happen to direct them.

"This view of the origin and nature of hysterical affections derives some confirmation from a circumstance which I have had frequent occasion to observe; although it has not, as far as I know, been noticed by pathological writers.—

In those who are much disposed to them, there is an evident weakness and laxity of the tissues, independently of what may be supposed to belong to the tissues of the nervous system. Thus there is a peculiar looseness of the joints; sometimes existing to such an extent that they are liable to a kind of subluxation (a slipping in and out, as the patient terms it), without any laceration of the synovial membrane or ligaments. I have known several cases in which a patient, on making some sudden exertion, has experienced a sensation as if some muscular or ligamentous fibres had given way; and, in some instances, a severe nervous pain, referred to this and the neighbouring parts, has remained for a long time afterwards. It is not unusual for the smaller blood-vessels to burst, so as to occasion slight hæmorrhage; although there is no actual disease in the bleeding part. This occurs most frequently with respect to the vessels of the mucous membranes. The disposition to hæmorrhage, however, is not peculiar to these textures. In a patient concerning whom I was consulted with Mr. Mawdsley, there had been repeated hæmorrhages from the ears.

These things must be regarded as indications of want of physical power in the system, and such is the prevailing character of hysterical disease; most distinctly marked, of course, in the most aggravated cases of the kind. A large proportion of hysterical patients suffer from cold hands and feet, have a feeble contracted pulse, a small appetite for food, and are wearied by very small exertions; they are more liable than other persons to lateral curvature of the spine. In some instances, and more especially in the parts which are most exposed to the external temperature, or at the greatest distance from the vital organs, the point of the nose, for example, and the ankles, the circulation is so weak that they assume at times a purple appearance, followed by vesications, and even by a thin slough." 72.

As Sir Benjamin remarks, these last-mentioned symptoms are a proof of an imperfect performance of the functions of the nervous system—at least they correspond to what is observed after injuries of the spinal chord, or even of the nerves.

When we pass to the treatment, it seems almost to follow as a consequence of the preceding observations, that much may be done by a well regulated system of physical and moral education, to mitigate the hysterical disposition in those who appear to inherit it, and to prevent its development in others, who are not so hereditarily and strongly predisposed to it.

To the medical profession is due the alleviation of what we might almost designate that penal code, under which young females were till lately educated. Physicians and surgeons have denounced so strongly the system of confining girls to heated rooms, of insisting on an inordinate quantum of study, and of debarring them from the enjoyment of the fresh air of heaven, and from that active exercise which nature has intended the young of both sexes to indulge in, that parents and teachers have taken the alarm, and a more rational as well as humane mode of discipline has been adopted.

Sir Benjamin Brodie insists on the propriety of medical men explaining to parents in the higher classes of society, the tendency of a strained and extravagant cultivation of the mental faculties, to engender hysteria in their female children. He justly observes that, after all, it is not cramming the mind with a large amount of facts or speculations, that can be regarded as the highest sort of mental culture. That is to be obtained by exercising the mind in right habits of thinking, and by enforcing the perception of those moral truths which display the necessity for self-control, and teach us how to practise it.

We fear, however, that constituted as society is, and as all civilized society must be, no system of education, no improvement of her health, no training of her mind, can be expected to do away with the tendency of the young female to hysterical disorders. They are too much dependent on the state of the uterus, and on the non-performance of its natural functions, to permit us to indulge the hope that hysterical disorders will not continue to afflict the sex which has to pay the tax of protracted and compulsory celibacy. If males were doomed, as females are, to pass the best years of their lives in absolute chastity, we doubt whether even their tougher nervous system would not often exhibit the convulsions of hysteria, or the thousand and one varieties of nervous symptoms, which we now see almost solely in woman.

Moral management is most effective in preventing the development of hysterical disorders; the physician or surgeon is seldom consulted until they are actually developed. The great principle of treatment then, consists in attempting to discover what organ or what function is disturbed, and in endeavouring to set that right. Nothing can be worse, yet unfortunately nothing is more common, than the empirical treatment of hysterical affections. One patient will have scanty menstruation, and all the signs of debility—another will be too plethoric—a third will have chronic inflammation of the uterus—a fourth will have little obvious physical derangement, and be only morally at fault. It surely cannot be right or rational to treat all these patients in the same way—to give them all the compound decoction of aloes or Griffith's mixture—to make them all take active exercise, or no exercise at all. Yet unfortunately, there are medical men who are too apt to look on hysteria, as hysteria, and to have one treatment for the one malady.

Perhaps the only remedy which is really applicable to every case is engagement of the mind. The more that can be diverted from brooding over real or imaginary miseries, the better the chance of the patient's recovery. It is not amusement only that is necessary—it is occupation that is requisite. A judicious combination of both will probably do more in the majority of cases, than all the drugs in the Pharmacopœia.

Sir Benjamin declines as trite and needless the consideration of the medical treatment of hysteria. What few suggestions he offers, will be found in the following extract.

"The whole class of tonic remedies, especially steel, quinine, sulphate of zinc, and ammonia, may, under certain circumstances, be employed with advantage. So also, it is of importance that the patient should live on a generous diet; that she should take exercise out of doors; that she should live in the pure air of the country rather than in that of a crowded city; and that her mind should be agreeably occupied, without being exhausted by great exertions. Nothing tends more to aggravate the disposition to hysteria than the tedium and *ennui* of a life without occupation; when the mind is, as it were, thrown back upon itself, brooding over imaginary misfortunes, and creating for itself objects of anxiety.

The use of what are usually called anti-spasmodic remedies, especially valerian and assafœtida, is indicated, not where there is merely a liability to hysterical symptoms, but where these symptoms are actually present. Those tonics which are useful in preventing these symptoms, are useful in the removal of them also, especially where the disease assumes a chronic form, as it generally does in the cases which fall under the observation of the surgeon. Here, also,

I have in several instances known much advantage to arise from a long-continued use of the sulphate of copper administered in pills, in small doses. Nor must we overlook another important rule of practice. There is often some particular circumstance in the state of the system at the time, which operates as the immediate exciting cause of the hysterical symptoms, and which medicine may remove. For example, in one individual there may be a furred tongue, and a costive state of the bowels; in another, deficient menstruation; and purgatives and emmenagogues may be administered with advantage, either separately or in combination. Again, it is not unusual in aggravated cases of hysteria to find the urine depositing a large quantity of lithic acid, in the form of sand; or the urine may be voided high-coloured, depositing a pink amorphous sediment, abounding in the lithate of ammonia; and in either of these cases the exhibition of alkalies, combined with alterative doses of mercury, purgatives, and a regulated diet, will contribute to produce a cure, the unhealthy quality of the urine seeming to be the cause, rather than the effect, of the hysterical affection." 77.

Sir Benjamin, no doubt from delicacy, makes no allusion to one remedy, more potent than all that have been mentioned—marriage. It is astonishing how quickly, under its influence, symptoms will disappear, which have baffled all management and medicine. When hysteria depends, as it usually does, on that state of system induced by constrained and unnatural celibacy, the due performance of the functions of the uterus might, à priori, be expected to lead to the restoration of health. In many instances, the rapidity of the effect is striking, and girls who seemed a prey to the most serious maladies are all at once transformed into healthy and happy women.

It must be owned, however, that mere sexual intercourse is not always sufficient to allay those nervous symptoms which continence mainly contributed to cause. The female was intended to become a mother; and if the uterus and the ovaria are prevented from playing the parts assigned to them in pregnancy, hysteria in its many shapes is again liable to be conjured up. In such a case, we must of course endeavour to ascertain the nature of the obstacle to conception and gestation, and having ascertained it, to remove it. A fruitful source both of hysteria and of barrenness is chronic inflammation of the uterus.

Sir Benjamin offers a few observations on the surgical treatment of local hysterical affections.

These are sometimes relieved by friction with a stimulating liniment—by the application of a belladonna plaister—by a spirituous lotion applied tepid*—by exposure of the part to the vapour of hot water, especially in cases of that peculiar affection of the wrist and hand which has been previously described.†

"In those cases in which the limb to which the symptoms are referred is affected alternately with heat and cold, I have known the following plan of treatment to be attended with excellent effects. During what may be termed the hot fit, let a compress be applied wet with a cold spirituous lotion; and when the heat has subsided, and the limb has become cold, let a thick woollen stocking be drawn over it, and then an oiled silk covering over the worsted stocking, so as

---

* ℞ Mist. camph. ℥iss.; Spir. rosmarini, ℥iss. M. ft. lotio.

† See the last number of this Journal.

to confine the heat and perspiration. When the cold fit has subsided, the oiled silk covering may be removed. This local treatment, however, should be combined with the exhibition of the sulphate of quinine, the use of which seems to be especially indicated by the intermitting character of the symptoms." 78.

Sir Benjamin deprecates local and general depletion, especially the latter. It may bring temporary alleviation—it entails ultimate debility and injury; for, what debilitates injures.

Blisters, issues, and counter-irritants of all sorts are open to one serious, indeed insuperable objection; they direct the patient's attention to the malady, while the object of the moral management is just the contrary. Thus, observes our author, in a case of hysterical neuralgia of the knee or hip, it seldom happens that any real amendment takes place while the patient remains confined as an invalid to her sofa. The pain may abate, but a sense of weakness follows, which disables her from walking more than the pain itself, and which, for obvious reasons, goes on increasing in proportion as the confinement is of longer duration. The first step towards a cure is, that she should have sufficient strength of mind to begin to use the limb in spite of present suffering.

A question of much interest naturally presents itself—Can these nervous symptoms be alleviated by cutting off the nervous communication of the part with the sensorium, or rather by cutting off its principal channels. When we turn to what has been done in tic-doulourex and tetanus, we find little to encourage us in operations on the nerves. When we reason on the causes of hysterical affections, we find still less to induce us to anticipate benefit from local operations. If a patient has violent pain in one limb, in consequence of uterine disturbance, what guarantee have we, that its removal by a serious operation from that limb, may not be followed by its re-appearance in another? It is clear that, from the nature of the case, no such warranty is to be got. Sir Benjamin relates a case in which he divided the nerves supplying the affected part, without, as we shall see, obtaining much benefit.

"In a case which I have already mentioned, of a young lady who had a train of most severe hysterical symptoms following the accidental prick of her finger, I was induced (many years ago) to divide the digital nerves. This was effected by a circular incision, carefully performed, extending through the whole of the nerves, integuments, vessels, and cellular texture, to the bones laterally, and to the aponeuroses of the tendons, anteriorly and posteriorly. The result was, that the patient's sufferings were aggravated rather than relieved." 81.

The same objections, in *principle*, lie against the total removal of the part affected. We mutilate a patient without being enabled to assure her that the next week may not bring with it the necessity for other mutilations still. Before we say more on this important subject, we will introduce the following cases, which our author has brought forward.

"As long ago as the year 1818, I was requested to visit a lady in the country on account of a disease of the knee. I was led to believe that she had laboured under an inflammation of the synovial membrane, which had in a great degree subsided, but that the harder textures had suffered in consequence, and that the cartilages were in danger of being ulcerated, and I recommended a plan of treatment accordingly. Whether, with my present experience on the subject, I should have taken the same view of her case, I will not undertake to say, but the result was, that a material improvement took place in the first instance. After some time, however, there was a manifest aggravation of all her symptoms. She suffered more than ever; so that she became anxious to undergo the amputation of the limb. I was now again consulted respecting her, but from the written accounts which I received, I concluded that the pain did not indicate the existence of any serious disease, and that the circumstances of the case did not justify so violent a measure as had been proposed. However, her wishes remained unaltered, and two surgeons of eminence in the country yielding to her entreaties, performed the operation. On dissection of the amputated joint, they were surprised to find that there was no collection of matter in its cavity; that the cartilages had disappeared in one spot, of very limited extent; and that there was no other mischief. The stump healed readily enough, but she obtained no relief. I had the opportunity of seeing her some months after the operation, suffering more than ever, with intense pain in the stump, and violent convulsive action of the muscles which move the thigh bone on the pelvis.

"Mr. Soden, of Bath, informed me of another of these cases, which fell under his observation, in which also the limb was amputated above the knee, but with no better result than in the case last mentioned. The symptoms attacked the stump, and the patient suffered as much after the operation as she had done before." 82.

So far as reasoning goes, amputation of a limb, or of any portion of the body, for 'hysterical' pain in it, is both absurd and cruel. It is absurd, because it does not remove the cause of the local malady; it is cruel, because it mutilates one of that sex to whom mutilation is a terrible calamity, and leaves her after all with every chance of suffering as much as she did before, in some remaining portion of her body. It is impossible, by any principle of reasoning or of common sense, to support the proposition, that when the fons et origo of these nervous disorders is in the uterine system, or in the general constitution of the nervous, amputation of a part to which pain may be *referred* can remove the cause of pain, that is, the disorder.

It may possibly be urged that, in many instances, we perform operations for the abstraction of parts which have suffered from diseases essentially constitutional in character. It may be said that we amputate a scrofulous joint, or a cancerous breast, and that therefore we may amputate a "hysterical" limb. The analogy is not a just one. The scrofulous articulation, or the scirrhous mamma has become in itself a serious disease, and the tissues are so altered that their restoration to a healthy condition is impossible. Nobody would dream of removing a knee in a scrofulous child, unless the bones, the cartilages, and soft parts were so disorganized as to be incompatible with recovery, however the original constitutional taint were done away with. Nay, so much has the conviction grown on surgeons, that operations are to be avoided in constitutional complaints, that even in these very instances of scrofula and cancer, they are now comparatively seldom had recourse to—in these very instances in which the effect becomes in a great measure independent of its cause, and where reason does not scout the barbarous folly of wielding the knife.

If it should be found that some surgeons warmly support the propriety of operating in certain cases of local hysterical affections, we may possibly suspect, that they are imperfectly acquainted with the general laws and phenomena of those disorders. We may imagine that they have taken a partial view of them, and fixing their attention on some prominent features, have lost the general bearing of the whole. If those surgeons appeal to empirical experience, and triumphantly refer to cases in which operations were successful, we may reasonably insist on a rigid scrutiny of all the circumstances, and we may endeavour to explain apparent success without abandoning those conclusions which seemed to follow from an accurate acquaintance with the malady.

1. Perhaps the most striking cases which have been adduced by those who advocate the propriety of operations are the following.

A. Mr. Mayo, in his Outlines of Pathology, has published the case of a young woman, whose knee he amputated on account of nervous pains. The stump healed. Soon after the stump was accidentally struck, and this slight accident was followed by pain in the part, exactly similar to that which had been referred formerly to the knee. Amputation was then performed a second time; but as the wound healed, the pain recurred, being again referred to the stump. Mr. Mayo then divided the sciatic nerve, below the edge of the *glutæus maximus* muscle. At first the pain was supposed to have been relieved, as after the former operations; but it returned on the wound being healed. Sir Benjamin at this time saw the patient, and the pain she endured was as severe as ever. Mr. Mayo, however, was not disheartened, nor was his patient at all disgusted with the knife. He removed the head of the thigh bone from the acetabulum, and up to May, 1836, she had had no recurrence of the pain. In the latter part of the Summer of that year we saw the patient, who applied to us for a ticket to the Margate Sea-Bathing Infirmary. It appeared that she had been under our own care in the first instance, that we had looked on the case as one of a hysterical description, and that we had discountenanced any very active measures. When she applied to us for a ticket for the Infirmary, she was apparently in good health, and declared that she had then no pain in the stump. But she had that expression of countenance so peculiar to hysterical patients, and in all probability from some very slight cause, or from no obvious cause at all, nervous symptoms might be set up, and be as violent as ever, in any part of the body to which accident should direct them. This, however, is conjecture. So far as the case at that time went, the *last* operation had relieved the patient.

B. "We are also informed," says Sir Benjamin Brodie, "that Sir Astley Cooper amputated the arm at the shoulder joint, on account of a neuralgic affection of a stump, and that the patient was permanently cured; and that a similar operation was performed successfully by Mr. Bransby Cooper. However, until we know more of these cases than is now recorded, it is impossible for us to determine whether they did or did not belong to the class of hysterical affections. Even if they did, the question still remains—how long did the patients remain under the observation of the surgeons afterwards—and was a cure really obtained, or was there simply a commutation of one hysterical affection for another?" 84.

Such are the main facts which have been cited in confirmation of the propriety of performing serious operations in cases of hysterical pains. Suppose we give them all the value claimed for them—suppose, for the sake of eliciting the truth, we look on them as really instances of cure. Are we to conclude that nature contradicts herself, and that while the whole history of these disorders seems to discountenance local operations, these are in reality of service? Or may we find that the contradiction is only apparent, and that the cures after operations may be explained without supposing them to have materially contributed to their occurrence? In order to solve questions of such obvious importance, Sir Benjamin offers the following considerations:—

"In estimating the value, not only of such operations, but of various other modes of treatment which have been supposed at one time or another to be useful in cases of aggravated hysteria, we are never to lose sight of the following circumstances:— 1. *Hysterical symptoms frequently disappear at once, without any manifest cause for their disappearance.* Examples of this fact may be found among the cases to which I have had occasion to refer in the preceding lectures. A young lady, who had been for more than two years confined to the recumbent posture on account of an hysterical affection simulating disease of the hip-joint, recovered suddenly one night while in the act of turning in bed. Another young lady, in whom a long train of most severe hysterical symptoms followed an accidental prick of one of her fingers, after the disease had existed for a great length of time (if I am not much mistaken, for more than two years), recovered also. 2. *It still more frequently happens that recovery from hysterical symptoms immediately follows a forcible impression of any kind made on the nervous system.* Hence it is, that any thing may obtain the credit of having effected a cure in these cases. Moral and physical agents are alike in this respect. Sometimes one remedy may appear to be successful, sometimes another; and that which is supposed to be productive of the greatest benefit in one case, may never be useful afterwards.

I have already mentioned the case of a young lady who, having long laboured under an hysterical neuralgia of the hip and thigh, rendering her unable to stand, or even to walk, immediately lost all her symptoms on being thrown from a donkey which she was riding; and the following are only a few among many other cases, which might be adduced in confirmation of what has been just stated.

In the eighth volume of the Transactions of the Royal Medical and Chirurgical Society, Mr. Pearson has described the case of a lady who laboured under a nervous affection of the hand and fore-arm, shewing itself in the form of severe pain and spasms of the muslces, and she immediately recovered on the application of a stimulating liniment, which, containing oil of turpentine, produced a vesicular eruption over the whole person.

I was informed, on good authority, of the case of a young lady who had long laboured under a severe hysterical affection, attended with spasmodic contraction of the muscles of one of the lower limbs, and which symptoms left her suddenly, on the extraction of a molar tooth.

Many years ago, I attended a young lady on account of a painful affection of the instep, which I certainly did not understand at the time, but of which, with my present experience on these subjects, I am satisfied that it was hysterical neuralgia, and nothing else. She was attended by other surgeons afterwards, who, I believe, were as much perplexed as I was, as to the nature of the disease, and who, at all events, gave her no relief. At last, while suffering as much as ever, she was informed of some remarkable cures obtained by the use of the vapour bath and champooing, and she immediately went to Brighton, that she might make a trial of these remedies. The first champooing gave her great relief; the second completed the cure. I was consulted respecting her afterwards, labouring under a nervous affection of the arm and fore-arm.

"In the 'Christian Observer' for November, 1830, we find recorded the case of Miss Fancourt, who had long been unable to move in consequence of what was evidently an hysterical affection, simulating disease of the hip-joint, and was supposed to have been miraculously cured under the influence of the prayers of her spiritual adviser, leaving her couch at once, and walking down stairs to supper, to the astonishment of her family." 87.

When we reflect on the facts which a close investigation of hysterical affections elicits—when we consider, *first*, that the nature of the disorder discountenances the idea, that any local remedy can effectually remove it: *secondly*, that positive experience has shewn the inutility of operations in many instances: *thirdly*, that hysterical symptoms suddenly disappear without any obvious cause for their departure: *fourthly*, that they are often forcibly removed by any powerful impression on the nervous system: and *fifthly*, that operations are not harmless remedies, which, if they do no good are attended with no evil—when we reflect upon all these circumstances, we may perhaps pronounce, with little risk of prophesying falsely, that no well-informed surgeons will employ the knife in these cases for the future.

With a parting observation of Sir Benjamin Brodie's we shall take our leave of this subject.

"We need not pursue this part of our inquiries further. To you who will soon be engaged in the practice of your profession, what I have now stated will be sufficient to impress your minds with a proper degree of scepticism, and to prevent you being misled by the caprices of these strange disorders. With respect to the great majority of society, whose minds are not accustomed to these investigations, and who do not know the difficulty of obtaining exact evidence as to the operation even of the remedies in common use, I feel that it will be almost a waste of time to endeavour to enlighten their minds on the subject. They will always be disposed to listen to, and to believe, the histories of the marvellous cures of hysterical affections—and with them conjurors of all kinds, from Prince Hohenlohe and the professors of animal magnetism, down to the most vulgar impostors, will always be the successful rivals of those practitioners who have studied their profession as a science." 88.

These few words contain some valuable hints for the profession, and a sarcastic estimate of public discernment. The grosser the imposture, the more greedily it is swallowed, both by titled and untitled fools, and the patronage of impostors shews the prevalence of ignorance, weakness, credulity, and fanaticism, in all classes of society, but more particularly in the higher. We are almost disposed to agree with our author, and to abandon in despair the attempt to reason on medical matters with a Public which has countenanced in untiring succession all sorts of quackery and quacks. Yet the fate of homœopathy may give us some encouragement, and induce us to believe that arguments, and appeals to their common sense, are not totally thrown away on the middle classes. The case of the aristocracy is hopeless.

In conclusion, we would strongly recommend these Lectures of Sir Benjamin Brodie's. The lessons which they read are valuable, and the practical surgeon who lays them to heart will find them of essential service in many an intricate case.

